# Leukocyte telomere length decreased the risk of mortality in patients with alcohol-associated liver disease

**DOI:** 10.3389/fendo.2024.1462591

**Published:** 2024-12-12

**Authors:** Jiahong Yi, Hui Guo, Chang Jiang, Junyi Duan, Ju Xue, Yue Zhao, Wenzhuo He, Liangping Xia

**Affiliations:** ^1^ Department of VIP Region, Collaborative Innovation Center for Cancer Medicine, State Key Laboratory of Oncology in South China, Guangdong Provincial Clinical Research Center for Cancer, Sun Yat-sen University Cancer Center, Guangzhou, China; ^2^ Collaborative Innovation Center for Cancer Medicine, State Key Laboratory of Oncology in South China, Guangdong Provincial Clinical Research Center for Cancer, Sun Yat-sen University Cancer Center, Guangzhou, China; ^3^ Department of Obstetrics and Gynecology, Zhuhai People’s Hospital, Zhuhai Hospital Affiliated with Jinan University, Zhuhai, China

**Keywords:** alcohol-associated liver disease, all-cause mortality, leukocyte telomere length, NHANES, prognosis

## Abstract

**Background:**

It is necessary to find latent indicators to predict the survival of alcohol-associated liver disease (ALD) patients. Leukocyte telomere length (LTL) was regarded as an indicator of prognosis in several diseases. However, the relationships between LTL and survival as well as cause-specific mortality in ALD patients were still unknown.

**Objective:**

This study aimed at exploring the underlying link between LTL and the risk of mortality in patients with ALD.

**Methods:**

The LTL and survival data were gathered from the National Health and Nutrition Examination Survey (NHANES) 1999–2002. The connection between LTL and mortality was assessed by Cox regression models and stratified analyses. The non-linear relationship was explored by restricted cubic spline (RCS) analysis. Sensitivity analyses were used to evaluate the robustness of our findings.

**Results:**

LTL was a negative factor for all-cause mortality (all *p*-value < 0.05). The risk of cardiovascular disease (CVD)-related death was decreased in Q3 (*p* < 0.001) and Q4 levels of LTL (*p* < 0.001) compared with the Q1 group. Shorter LTL resulted in higher cancer-caused mortality (*p* = 0.03) in the Q2 group. Longer LTL improved survival especially for elder patients (*p* for trend < 0.001) or men (*p* for trend = 0.001). Moreover, there were L-shaped correlations between LTL and all-cause mortality (*p* for non-linearity = 0.02), as well as cancer-related mortality (*p* for non-linearity < 0.001). Four sensitivity analyses proved the robustness of our findings.

**Conclusion:**

Our research found that longer LTL improved survival in patients with ALD and decreased CVD and cancer-related mortality. LTL decreased all-cause mortality especially for patients older than 65 years or men. LTL might be a useful biomarker for prognosis among patients with ALD. More prospective studies are needed to assess the relevance between LTL and mortality and explore the underlying mechanisms between them.

## 1 Introduction

Alcohol-related liver disease (ALD) is a common type of liver condition, responsible for approximately a quarter of cirrhosis-related deaths. There are approximately 2.18 million patients with ALD in the United States (1). Approximately 10% of ALD patients experienced further aggravation of their cirrhosis (2, 3). Furthermore, ALD accounts for a large number of patients who received liver transplantation in the United States (4). ALD has a poor prognosis, and symptoms, such as jaundice, hepatorenal syndrome, and hepatopulmonary syndrome, usually occur at the last stage (5, 6). The mortality of ALD is currently 1.75 times higher than 20 years ago in the United States (7). Because of this, it is necessary to find biomarkers for predicting the survival of ALD patients. Though several imaging techniques and prognostic models have provided pertinent evidence to assess the process of ALD, it still remains controversial (8–11).

Telomeres are located at the end of the chromosomes and their function is to maintain DNA stability (12). Generally, telomeres become shorter as the cells divide. When the length becomes extremely short, cells will become senescent or apoptotic (13). Thereby, leukocyte telomere length (LTL) was regarded as an index of the biological aging process of cells, and it is a common phenomenon occurring in any cellular tissues (14). LTL reflected the senescent status of circulating immune cells (12). LTL was reported to be related to many diseases, such as multiple sclerosis (MS), malignant neoplasms, and cardiovascular diseases (CVDs) (15–19). Longer LTL decreased the risk of NAFLD incidence and mediated the link between age and NAFLD in the UK Biobank (20). Another recent Mendelian randomization study illustrated that there was no clear association between LTL and ALD (21). Nevertheless, we still do not know how LTL affects prognosis in patients with ALD. LTL was related to advanced liver disease among old people, and it had a negative relationship with all-cause mortality in patients with liver disease (22). Furthermore, the level of LTL was considered as a symbol of all-cause mortality in certain populations (23, 24). Thereby, the telomere length of leukocytes had a positive influence on the prognosis of age-related diseases (25–28). However, it remains uncertain whether LTL was a prospective predictor of survival and cause-specific death in individuals with ALD.

Therefore, we hypothesized that LTL might correlate with all-cause and cause-specific mortality in ALD patients. Our study was aimed at exploring the latent link between LTL and all-cause and cause-specific mortality in patients with ALD from the United States so that high-risk patients can take effective measures to improve their survival.

## 2 Methods

### 2.1 Study population

The data in this retrospective research were sourced from NHANES 1999–2002 and were acquired from the website https://www.cdc.gov/nchs/nhanes/index.htm. Every enrolled participant was asked to provide an informed consent in written form. The National Center for Health Statistics Research Ethics Review Board (NCHS ERB) approved, censored, and ratified the program.

**Figure 1:**
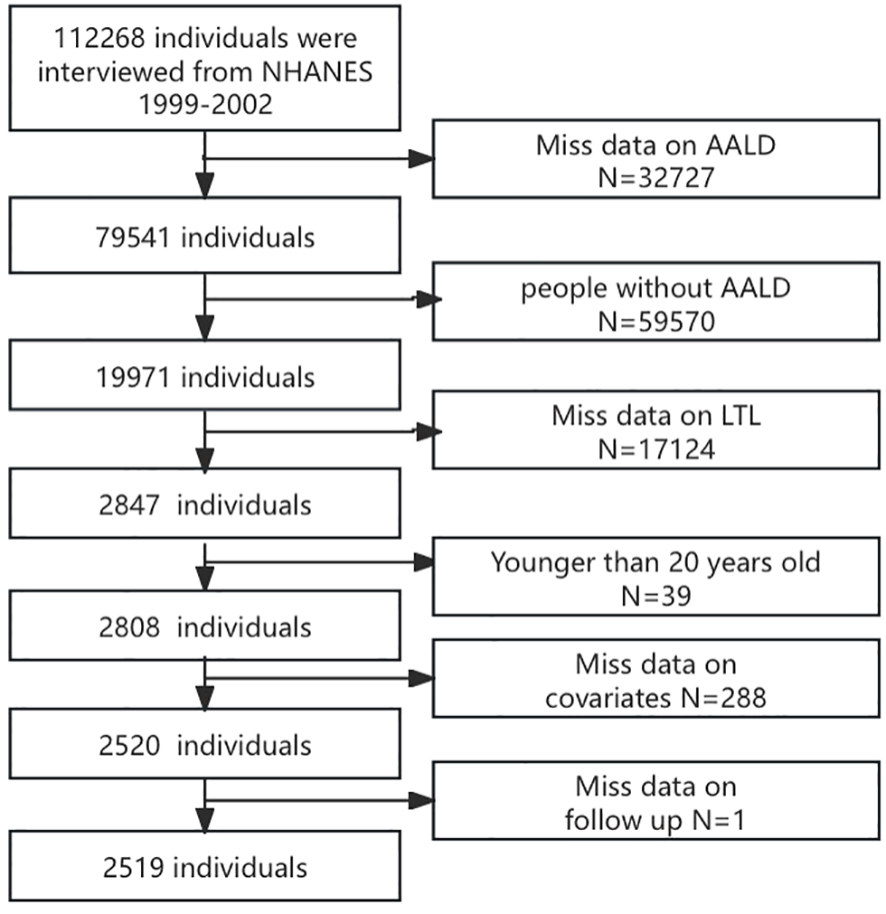
Flowchart of the population in this study.

After screening, data from 2,519 participants after screening were analyzed based on specific inclusion and exclusion criteria. The exclusion criteria were as follows: (a) did not have ALD, (b) missing data on LTL, (c) younger than 20 years, (d) missing data on covariates, and (e) follow-up data. The detailed selection procedure is displayed in **Figure 1**.

### 2.2 ALD status

ALD was identified according to a previous study. In summary, adults were classified as ALD if they met the following criteria: (1) consumed over 28 g of alcohol daily for women and over 42 g for men in the past year, (2) had high levels of liver enzymes such as AST and ALT, and (3) did not have HCV/HBV or NAFLD (29).

### 2.3 Study variables

In the “laboratory” portion of the NHANES interview, LTL was collected. Polymerase chain reaction was used to evaluate the telomere length. The relative ratio against standard reference DNA (T/S) was determined through calculation. The base pairs were modified for analysis using information based on previous research (12, 24). The detailed information was shown on the NHANSE website.

### 2.4 Other covariates

From the demographic information section, we gathered data on age, gender, race, marital status, level of education, the income-to-poverty ratio (PIR), and smoking habits. Data on albumin (g/L), C-reactive protein (CRP, mg/dL), alanine aminotransferase (ALT, U/L), aspartate transaminase (AST, U/L), alkaline phosphatase (ALP, U/L), total bilirubin (mg/dL), and lactate dehydrogenase (LDH, U/L) were gathered from the examination information section and used as indicators of liver function. The history of hypertension, DM, and other comorbidities was based on questionnaire data.

### 2.5 Follow-up data

The follow-up of this study ended on 31 December 2019. Death from all reasons was defined as all-cause mortality. Other causes such as CVD (I00–I09, I11, I13, I20–I51, and I60–I69), cancer (C00–C97), DM (E10–E14), chronic lower respiratory diseases (J40–J47), Alzheimer’s disease (G30), nephritis, nephrotic syndrome, and nephrosis (N00–N07, N17–N19, and N25–N27), accidents (unintentional injuries) (V01–X59 and Y85–Y86), and influenza and pneumonia (J09–J18) were categorized according to the International Statistical Classification of Diseases and Related Health Issues (ISCDRH).

### 2.6 Statistical methods

Sample weights were considered in our study in accordance with the NCHS analytic guidelines. All methods in this study were performed in accordance with the relevant guidelines and regulations in the NHANES website. Continuous variables were described as means ± standard errors and categorical variables were shown as number (percentage), respectively. We divided TLT into quadrants; the ranges of LTL in Q1, Q2, Q3, and Q4 were (0.39,0.834], (0.83,0.98], (0.98,1.16], and (1.157,9.42], respectively. *t*-test, Mann–Whitney *U* test, and chi-square test were employed for comparing normally distributed data, non-normally distributed continuous data, and categorical variables, respectively. The association between LTL and all-cause or cause-specific mortality was explored by four Cox regression models. No adjustments for covariates were made in Model 1. Model 2 was modified to account for age, gender, and race. Model 3 was additionally modified by taking into account education, smoke, hypertension, and diabetes, building upon the adjustments made in Model 2. Model 4 was calibrated for additional laboratory parameters, such as albumin, CRP, ALT, AST, ALP, total bilirubin, and LDH. Furthermore, prognostic differences among the four levels of TLT were estimated using Kaplan–Meier analysis and the log-rank test. The non-linear relationship was evaluated by restricted cubic spline (RCS) analyses. Stratified analysis was carried out to assess the variances among different subgroups. Finally, the robustness of our findings was evaluated by performing four sensitivity analyses in this research. The first and second analyses were carried out after excluding individuals who died within 1 year or 2 years of follow-up. The third analysis was conducted after excluding individuals with cancer. The fourth analysis did not adjust for NHANES survey weights.

The analyses mentioned above were conducted using R (version 4.3.3) and R Studio. The R packages “rms”, “survey”, and “ggplot2” were used. *p*-value less than 0.05 was deemed to be statistically significant.

## 3 Results

### 3.1 Baseline characteristics of included patients in NHANES 1999–2002

Among the 2,519 enrolled ALD patients, 409 (9.04%) were older than 65 years and 2,110 (90.96%) were younger. There were 1,775 (69.02%) men. A total of 1,689 (82.70%) patients were Non-Hispanic white or black, 648 (7.40%) were Mexican American, and 182 (9.90%) patients were from other races or were multiracial. A total of 928 (31.71%) individuals had hypertension, and 257 (7.14%) had diabetes. Patients with a higher level of LTL seemed younger (*p* < 0.001). Fewer patients had hypertension (*p* < 0.001) or DM (*p* < 0.001) in the higher LTL group. Moreover, CRP (*p* < 0.001), ALP (*p* = 0.004), and LDH (*p* = 0.002) tended to decrease in higher levels of LTL, as shown in **Table 1**. We further analyzed whether LTL was related to comorbidity profile among patients with ALD. Except for hypertension or DM, patients with longer LTL had a lower risk of chronic kidney disease, CVD, peripheral arterial disease, and metabolic syndrome (all *p* < 0.05), as shown in **Supplementary Table 1**.

**Table 1:** Baseline characteristics of included patients in NHANES 1999–2002 ^a^.

Variables	Total	Q1	Q2	Q3	Q4	*p*-value
Age (years)						<0.001
>65	409 (9.04%)	211 (21.42%)	109 (9.67%)	59 (5.36%)	30 (2.86%)	
≤65	2,110 (90.96%)	418 (78.58%)	521 (90.33%)	572 (94.64%)	599 (97.14%)	
Gender						0.5
Male	1,775 (69.02%)	451 (67.75%)	465 (71.54%)	423 (67.11%)	436 (69.50%)	
Female	744 (30.98%)	178 (32.25%)	165 (28.46%)	208 (32.89%)	193 (30.50%)	
Race						0.59
Non-Hispanic white or black	1,689 (82.70%)	426 (84.07%)	411 (83.70%)	411 (82.31%)	441 (81.21%)	
Mexican American	648 (7.40%)	162 (6.36%)	179 (8.23%)	171 (8.00%)	136 (6.92%)	
Other race/multiracial	182 9.90%)	41 (9.56%)	40 (8.08%)	49 (9.69%)	52 (11.87%)	
Marital status						<0.001
Divorced or separated or widowed	409 (14.51%)	134 (17.98%)	103 (15.56%)	94 (13.62%)	78 (11.92%)	
Married	1,647 (65.32%)	439 (72.48%)	437 (67.88%)	406 (64.62%)	365 (58.60%)	
Single	342 (14.54%)	41 (6.71%)	65 (11.99%)	102 (16.58%)	134 (20.53%)	
Unknow	121 (5.63%)	15 (2.83%)	25 (4.57%)	29 (5.18%)	52 (8.95%)	
Education						0.89
Less than high school	718 (17.56%)	208 (20.12%)	182 (17.64%)	174 (17.24%)	154 (15.91%)	
College graduate or above	566 (27.74%)	129 (26.34%)	143 (27.14%)	151 (28.69%)	143 (28.39%)	
High school grad/GED or equivalent	580 (25.66%)	137 (24.54%)	131 (25.21%)	144 (25.44%)	168 (27.05%)	
Other	655 (29.05%)	155 (29.00%)	174 (30.02%)	162 (28.62%)	164 (28.65%)	
Smoker						0.07
No	1,102 (43.99%)	250 (39.02%)	273 (42.18%)	289 (48.08%)	290 (45.43%)	
Yes	1,417 (56.01%)	379 (60.98%)	357 (57.82%)	342 (51.92%)	339 (54.57%)	
Hypertension						<0.001
No	1,591 (68.29%)	346 (58.88%)	375 (65.46%)	414 (69.21%)	456 (76.65%)	
Yes	928 (31.71%)	283 (41.12%)	255 (34.54%)	217 (30.79%)	173 (23.35%)	
DM						<0.001
No	2,142 (89.19%)	505 (84.64%)	516 (86.29%)	554 (91.72%)	567 (92.64%)	
IFG	120 (3.67%)	40 (5.84%)	32 (4.62%)	30 (3.08%)	18 (1.83%)	
DM	257 (7.14%)	84 (9.51%)	82 (9.09%)	47 (5.20%)	44 (5.52%)	
PIR	3.30 (0.08)	3.39 (0.12)	3.41 (0.10)	3.23 (0.11)	3.19 (0.11)	0.23
Albumin (g/L)	44.56 (0.14)	44.09 (0.23)	44.47 (0.24)	44.63 (0.16)	44.91 (0.19)	0.05
CRP (mg/dL)	0.36 (0.01)	0.50 (0.04)	0.38 (0.04)	0.34 (0.02)	0.28 (0.02)	<0.001
ALT (U/L)	34.60 (0.67)	34.23 (1.38)	36.39 (1.51)	33.71 (1.16)	34.15 (1.08)	0.06
AST (U/L)	28.95 (0.58)	29.37 (1.26)	29.13 (0.92)	28.21 (0.59)	29.16 (1.27)	0.59
ALP (U/L)	74.86 (1.15)	77.24 (1.69)	76.50 (1.42)	73.92 (1.47)	72.61 (1.41)	0.004
Total Bilirubin (mg/dL)	0.70 (0.01)	0.67 (0.02)	0.68 (0.01)	0.72 (0.02)	0.71 (0.01)	0.03
LDH (U/L)	143.21 (1.13)	148.23 (2.04)	143.67 (2.81)	139.85 (1.49)	142.20 (1.97)	0.002

a Data were adjusted for NHANES survey weights. Q1, quartile 1; Q2, quartile 2; Q3, quartile 3; Q4, quartile 4; DM, diabetes mellitus; PIR, household poverty-to-income ratio; CRP, C-reactive protein; ALT, alanine aminotransferase; AST, aspartate transaminase; ALP, alkaline phosphatase; LDH, lactate dehydrogenase, IFG, impaired fasting glucose.

The p-values in bold denote statistical significance.

In addition, a total of 552 (15.49%) patients died at the end of follow-up. Mortality was the highest in the Q1 group, which had the lowest level of LTL (*p* < 0.001). Causes of death included accidents (4.30%), Alzheimer’s disease (4.52%), cancer (26.45%), CVD (28.99%), DM (3.26%), influenza or pneumonia (2.72%), kidney diseases (1.81%), chronic lower respiratory diseases (4.90%), and other causes (23.00%). The percentage of mortality was the highest in the Q4 group, suggesting that there was a relationship between higher LTL and decreased risk of death in patients with ALD (**Table 2**).

**Table 2:** Mortality distribution among ALD patients ^a^.

Variables	Total	Q1	Q2	Q3	Q4	*p*-value
Survival status						<0.001
Live	1,967 (84.51%)	386 (71.76%)	496 (83.78%)	529 (87.55%)	556 (91.62%)	
Dead	552 (15.49%)	243 (28.24%)	134 (16.22%)	102 (12.45%)	73 (8.38%)	
Causes of death						<0.001
Accidents	24 (4.30%)	11 (20%)	4 (0.72%)	5 (0.91%)	4 (0.72%)	
Alzheimer's disease	25 (4.52%)	12 (2.17%)	7 (1.27%)	3 (0.54%)	3 (0.54%)	
Cancer	146 (26.45%)	63 (11.41%)	26 (4.71%)	33 (6.00%)	24 (4.35%)	
CVD	160 (28.99%)	70 (12.68%)	44 (7.97)	30 (5.43%)	16 (2.90%)	
DM	18 (3.26%)	4 (0.72%)	4 (0.72%)	4 (0.72%)	6 (1.09%)	
Influenza and pneumonia	15 (2.72%)	9 (1.63%)	2 (0.36%)	2 (0.36%)	2 (0.36%)	
Kidney disease	10 (1.81%)	5 (0.91%)	5 (0.91%)	0 (0.00%)	0 (0.00%)	
Chronic lower respiratory diseases	27 (4.90%)	14 (2.54%)	9 (1.63%)	2 (0.36%)	2 (0.36%)	
Other causes	127 (23.00%)	55 (10.00%)	33 (6.00%)	23 (4.17%)	16 (3.00%)	

a Data were adjusted for NHANES survey weights. Q1, quartile 1; Q2, quartile 2; Q3, quartile 3; Q4, quartile 4; CVD, cardiovascular disease; DM, diabetes mellitus.

The p-values in bold denote statistical significance.

### 3.2 The association between telomere length and all-cause mortality in ALD patients

**Table 3** illustrates the association between LTL and all kinds of mortality. Longer LTL reduced all-cause mortality in Q2 (HR = 0.54 [0.42–0.71], *p* < 0.001), Q3 (HR = 0.26 [0.14–0.48], *p* < 0.001), and Q4 (HR = 0.12 [0.06–0.25], *p* < 0.001) compared with the Q1 group in Model 1. In Model 2, LTL remained a protective factor with HRs and 95% CIs of 0.72 [0.55–0.96], 0.64 [0.45–0.90], and 0.46 [0.33–0.64] in the Q2, Q3, and Q4 groups (all *p*-value < 0.05), respectively. The difference still existed in Model 3 and Model 4.

**Table 3:** The association of LTL with all-cause mortality ^a^.

Character	Model 1			Model 2			Model 3			Model 4		
	HR	95% CI	*p*-value	HR	95% CI	*p*-value	HR	95% CI	*p*-value	HR	95% CI	*p*-value
Q1	–	–	–	–		–	–		–	–		–
Q2	0.54	0.42–0.71	**<0.001**	0.72	0.55–0.96	**0.02**	0.7	0.54–0.91	**0.01**	0.72	0.56–0.94	**0.01**
Q3	0.4	0.30–0.55	**<0.001**	0.64	0.45–0.90	**0.01**	0.63	0.46–0.88	**0.01**	0.68	0.48–0.96	**0.03**
Q4	0.27	0.20–0.36	**<0.001**	0.46	0.33–0.64	**<0.001**	0.49	0.35–0.69	**<0.001**	0.52	0.37–0.73	**<0.001**

a Data were adjusted for NHANES survey weights. Model 1 was not adjusted for any covariates. Model 2 was adjusted for age, gender, and race. Model 3 was further adjusted for education, smoke, hypertension, and DM on the basis of Model 2. Model 4 was adjusted for other laboratory indicators, including PIR, albumin (g/L), CRP (mg/dL), ALT (U/L), AST (U/L), ALP (U/L), total bilirubin (mg/dL), and LDH (U/L). Q1, quartile 1; Q2, quartile 2; Q3, quartile 3; Q4, quartile 4; HR, hazard ratio; CI, confidence interval.

The p-values in bold denote statistical significance.

**Figure 2:**
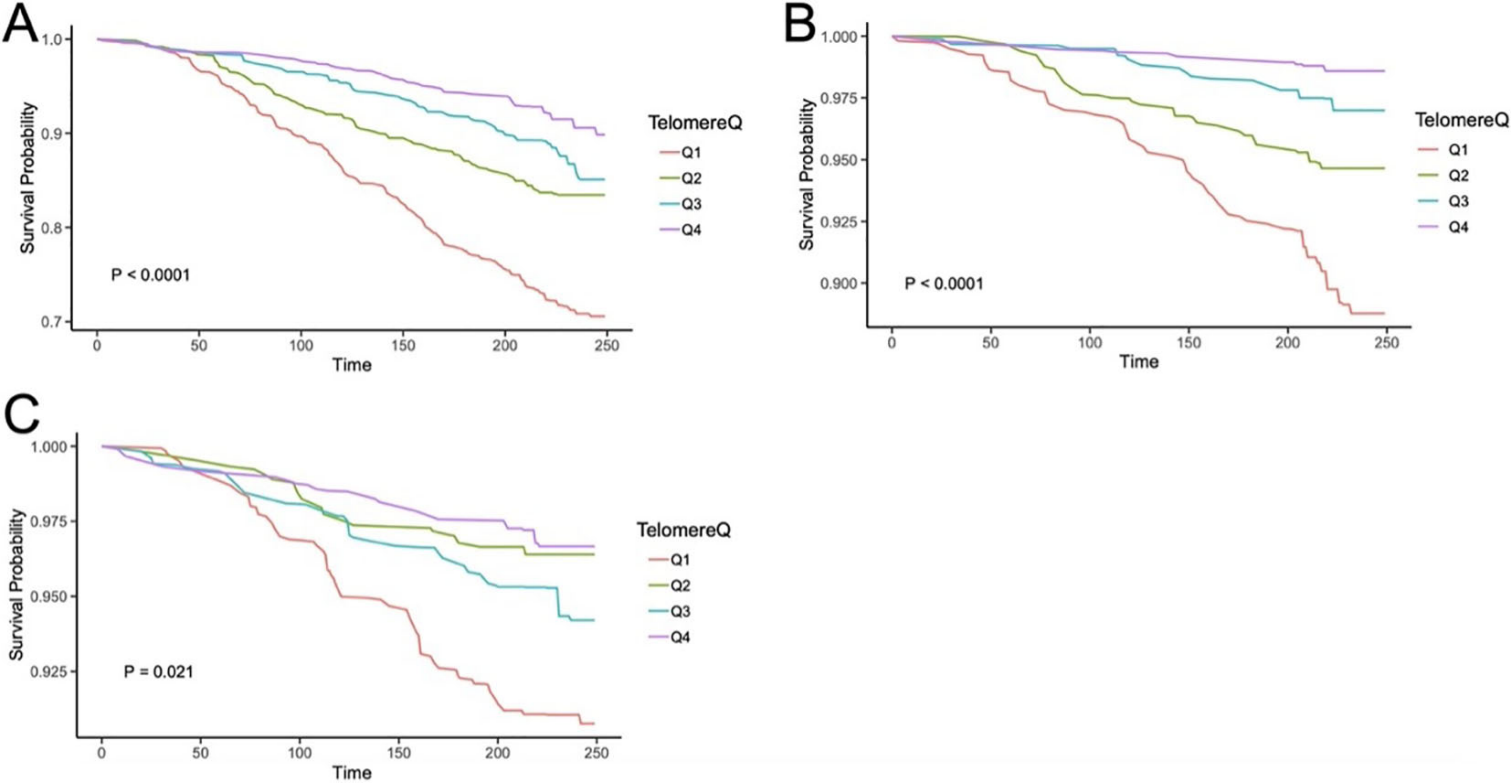
The Kaplan–Meier curves for LTL associating with all-cause mortality **(A)**, CVD mortality **(B)**, and cancer-caused mortality **(C)** in ALD patients. Q1, quartile 1; Q2, quartile 2; Q3, quartile 3; Q4, quartile 4; CVD, cardiovascular disease.

Consistently, LTL remained negatively associated with all-cause mortality when it was regarded as a continuous variable (**Supplementary Table 2**). **Figure 2A** shows the Kaplan–Meier survival curve (*p* < 0.001).

### 3.3 The correlation between LTL and cause-specific mortality in patients with ALD

As regards mortality caused by cardiovascular disease, LTL decreased the risk of death in the crude model (*p* < 0.001 for all groups) compared to the Q1 group. In Model 2, both the Q3 and Q4 groups had lower HRs (0.40 [0.23–0.67] for Q3, *p* < 0.001; 0.2 [0.1–0.3] for Q4, *p* < 0.001) compared with the Q1 group. The results in Model 3 and Model 4 were consistent with Model 2, as indicated in **Table 4**.

**Table 4:** The association of LTL with cause-specific mortality ^a^.

Causes of death	Model 1			Model 2			Model 3			Model 4		
	HR	95% CI	*p*-value	HR	95% CI	*p*-value	HR	95% CI	*p*-value	HR	95% CI	*p*-value
CVD												
Q1	–	–		–	–		–	–		–	–	
Q2	0.5	0.34–0.73	**<0.001**	0.65	0.41–1.02	0.06	0.65	0.40–1.04	0.07	0.65	0.41–1.05	0.08
Q3	0.26	0.14–0.48	**<0.001**	0.4	0.23–0.67	**<0.001**	0.42	0.25–0.70	**0.001**	0.45	0.27–0.76	**0.003**
Q4	0.12	0.06–0.25	**<0.001**	0.2	0.10–0.39	**<0.001**	0.22	0.11–0.44	**<0.001**	0.23	0.12–0.44	**<0.001**
Cancer												
Q1	–		–	–		–	–		–	–		–
Q2	0.39	0.20–0.76	**0.01**	0.47	0.25–0.89	**0.02**	0.5	0.28–0.91	**0.02**	0.51	0.28–0.95	**0.03**
Q3	0.56	0.31–1.02	0.06	0.79	0.44–1.41	0.42	0.86	0.48–1.54	0.62	0.93	0.52–1.66	0.8
Q4	0.34	0.18–0.67	**0.002**	0.51	0.27–0.94	**0.03**	0.55	0.28–1.10	0.09	0.6	0.30–1.17	0.13

a Data were adjusted for NHANES survey weights. Model 1 was not adjusted for any covariates. Model 2 was adjusted for age, gender, and race. Model 3 was further adjusted for education, smoke, hypertension, and DM on the basis of Model 2. Model 4 was adjusted for other laboratory indicators, including PIR, albumin (g/L), CRP (mg/dL), ALT (U/L), AST (U/L), ALP (U/L), total bilirubin (mg/dL), and LDH (U/L). Q1, quartile 1; Q2, quartile 2; Q3, quartile 3; Q4, quartile 4; HR, hazard ratio; CI, confidence interval; CVD, cardiovascular disease.

The p-values in bold denote statistical significance.

In terms of cancer-related mortality, Q2 (HR = 0.39 [0.20–0.76], *p* = 0.013) and Q4 (HR = 0.34 [0.18–0.67], *p* = 0.002) had decreased HRs compared to the Q1 group in Model 1. For Model 2, the cancer-caused death decreased in the Q2 group (HR = 0.47 [0.25–0.89], *p* = 0.02) and the Q4 group (HR = 0.51 [0.27–0.94], *p* = 0.03), but not in the Q3 group (HR = 0.79 [0.44–1.40], *p* = 0.42). The negative correlation between LTL and mortality was still evident in the Q2 group according to Model 3 (HR = 0.5 [0.28–0.91], *p* = 0.02) or Model 4 (HR = 0.51 [0.28–0.95], *p* = 0.03).

When LTL was considered as a continuous factor, longer LTL was a protective factor from CVD mortality in the four models, while cancer-caused mortality declined with extended LTL only in Model 1 (see **Supplementary Table 2**). Their Kaplan–Meier survival curves are shown in **Figures 2B, C** (p values were < 0.001 for CVD death and 0.021 for cancer-caused death).

### 3.4 The correlation between LTL and mortality in various subgroups

Stratified analysis showed that longer LTL decreased all-cause mortality regardless of smoking status, hypertension, or DM (all *p* for trend < 0.05) among patients with ALD. As shown in **Table 5**, LTL was a positive factor for survival especially for elder patients (*p* for trend < 0.001) or men (*p* for trend = 0.001).

**Table 5:** The association between dietary iron intake and all-cause mortality in different subgroups ^a^.

Character	Q1	Q2	*p*-value	Q3	*p*-value	Q4	*p*-value	*p* for trend
Age (years)								
≤65	–	0.83 (0.58–1.18)	0.29	0.85 (0.53–1.37)	0.52	0.67 (0.43–1.05)	0.08	0.13
>65	–	0.67 (0.50–0.92)	**0.01**	0.60 (0.39–0.90)	**0.01**	0.32 (0.18–0.60)	**<0.001**	**<0.001**
Gender								
Male	–	0.83 (0.64–1.08)	0.17	0.73 (0.50–1.07)	0.11	0.53 (0.37–0.78)	**0.001**	**0.001**
Female	–	0.49 (0.22–1.14)	0.1	0.56 (0.30–1.08)	0.08	0.56 (0.31–1.02)	0.06	0.07
Smoker								
No	–	0.60 (0.36–1.00)	**0.05**	0.59 (0.36–0.97)	**0.04**	0.51 (0.28–0.92)	**0.02**	**0.01**
Yes	–	0.78 (0.58–1.05)	0.1	0.70 (0.48–1.04)	0.08	0.52 (0.35–0.77)	**0.001**	**0.002**
Hypertension								
No	–	0.82 (0.54–1.23)	0.34	0.63 (0.39–1.02)	0.06	0.57 (0.33–0.99)	**0.05**	**0.03**
Yes	–	0.67 (0.45–1.00)	**0.05**	0.78 (0.51–1.20)	0.26	0.52 (0.32–0.86)	**0.01**	**0.03**
DM								
No	–	0.78 (0.52–1.17)	0.24	0.75 (0.47–1.20)	0.23	0.57 (0.37–0.87)	**0.01**	**0.02**
IFG	–	0.71 (0.25–2.04)	0.52	0.72 (0.31–1.66)	0.45	0.09 (0.01–0.56)	**0.01**	**0.03**
DM	–	0.41 (0.24–0.70)	**0.001**	0.36 (0.20–0.66)	**<0.001**	0.55 (0.27–1.10)	0.09	**0.03**

a Data were adjusted for NHANES survey weights. The stratified analysis was adjusted for age, gender, race, education, smoke, hypertension, DM, PIR, albumin (g/L), CRP (mg/dL), ALT (U/L), AST (U/L), ALP (U/L), total bilirubin (mg/dL), and LDH (U/L). Q1, quartile 1; Q2, quartile 2; Q3, quartile 3; Q4, quartile 4; HR, hazard ratio; CI, confidence interval; IFG, impaired fasting glucose; DM, diabetes mellitus.

The p-values in bold denote statistical significance.

### 3.5 The non-linear relationship between LTL and mortality in ALD patients

**Figure 3:**
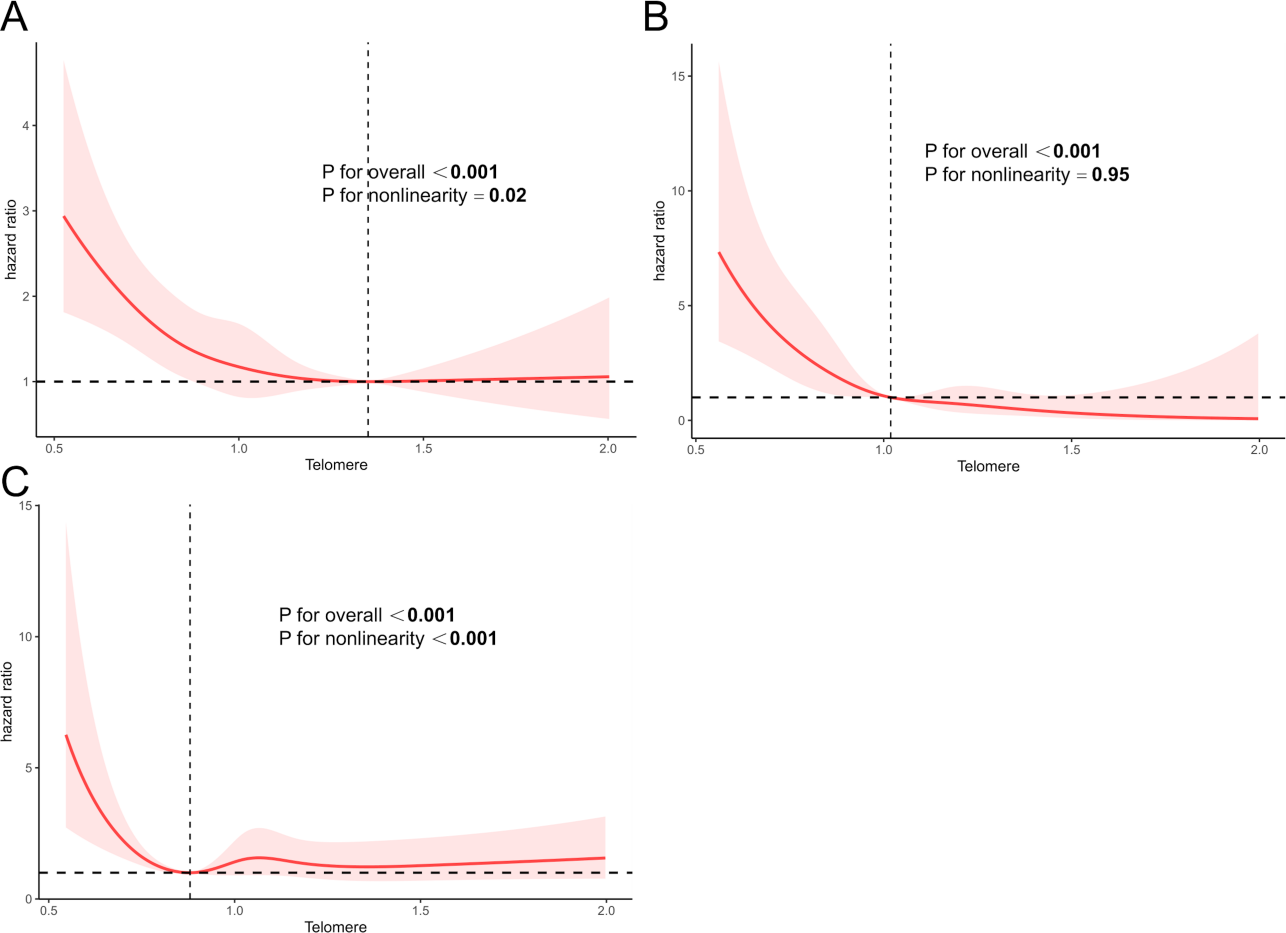
The RCS curve between LTL with all-cause mortality **(A)**, CVD mortality **(B)**, and cancer-caused mortality **(C)** in participants with ALD. Q1, quartile 1; Q2, quartile 2; Q3, quartile 3; Q4, quartile 4; CVD, cardiovascular disease. The RCS models were adjusted for age, gender, race, education, smoke, hypertension, DM, and other laboratory indicators, such as PIR, albumin (g/L), CRP (mg/dL), ALT (U/L), AST (U/L), ALP (U/L), total bilirubin (mg/dL), and LDH (U/L).

The significant non-linear relationship between LTL and all-cause mortality is demonstrated in **Figure 3A**. The curve appeared L-shaped (***p*** for non-linearity = 0.02). All-cause mortality decreased sharply when LTL increased to 1.36 and tended to flatten subsequently. LTL did not exhibit a non-linear correlation with CVD mortality (*p* for non-linearity = 0.95) (**Figure 3B**). When it came to cancer-related mortality, the L-shaped curve was also found to have a tendency to decrease mortality and LTL (*p* for non-linearity < 0.001) (**Figure 3C**).

### 3.6 Sensitivity analyses

Sensitivity analyses were conducted to evaluate the robustness of our findings. The findings remained robust after excluding individuals who died within 1 year (**Table 6**) or 2 years of follow-up (**Supplementary Table 3**), excluding individuals with cancer (**Supplementary Table 4**), or not adjusting for NHANES survey weights (**Supplementary Table 5**). All-cause mortality and CVD-related mortality still declined with higher levels of LTL. Though there was no statistical significance for cancer-caused death in some sensitivity analyses, HR still declined in higher levels of LTL compared with the lowest group.

**Table 6:** The association between LTL and mortality after excluding individuals who died within 1-year follow-up ^a^.

Character	Model 1		Model 2		Model 3		Model 4	
	HR (95% CI)	*p*-value	HR (95% CI)	*p*-value	HR (95% CI)	*p*-value	HR (95% CI)	*p*-value
All causes								
Q1	–	–	–	–	–	–	–	–
Q2	0.55 (0.42–0.72)	**<0.0001**	0.73 (0.55–0.97)	**0.03**	0.70 (0.54–0.91)	**0.01**	0.73 (0.56–0.94)	**0.02**
Q3	0.40 (0.29–0.55)	**<0.0001**	0.63 (0.44–0.90)	**0.01**	0.63 (0.45–0.88)	**0.01**	0.67 (0.47–0.96)	**0.03**
Q4	0.26 (0.19–0.35)	**<0.0001**	0.45 (0.32–0.62)	**<0.0001**	0.48 (0.34–0.67)	**<0.0001**	0.51 (0.37–0.70)	**<0.0001**
CVD								
Q1	–	**-**	–	–	–	–	–	–
Q2	0.50 (0.34–0.75)	**<0.0001**	0.66 (0.41–1.05)	0.08	0.66 (0.41–1.06)	0.09	0.66 (0.41–1.07)	0.09
Q3	0.26 (0.14–0.49)	**<0.0001**	0.39 (0.22–0.69)	**0.001**	0.41 (0.24–0.72)	**0.002**	0.44 (0.25–0.77)	**0.004**
Q4	0.13 (0.06–0.25)	**<0.0001**	0.20 (0.10–0.40)	**<0.0001**	0.22 (0.11–0.45)	**<0.0001**	0.23 (0.12–0.46)	**<0.0001**
Cancer								
Q1	–	–	–	–	–	–	–	–
Q2	0.39 (0.20–0.76)	**0.01**	0.47 (0.25–0.89)	**0.02**	0.51 (0.28–0.91)	**0.02**	0.51 (0.28–0.94)	**0.03**
Q3	0.56 (0.31–1.02)	0.06	0.79 (0.44–1.42)	0.44	0.87 (0.49–1.56)	0.64	0.94 (0.52–1.68)	0.83
Q4	0.31 (0.16–0.61)	**<0.0001**	0.46 (0.24–0.86)	**0.02**	0.50 (0.26–0.97)	**0.04**	0.53 (0.28–1.03)	0.06

a Data were adjusted for NHANES survey weights. Model 1 was not adjusted for any covariates. Model 2 was adjusted for age, gender, and race. Model 3 was further adjusted for education, smoke, hypertension, and DM on the basis of Model 2. Model 4 was adjusted for other laboratory indicators, including PIR, albumin (g/L), CRP (mg/dL), ALT (U/L), AST (U/L), ALP (U/L), total bilirubin (mg/dL), and LDH (U/L). Q1, quartile 1; Q2, quartile 2; Q3, quartile 3; Q4, quartile 4; HR, hazard ratio; CI, confidence interval; CVD, cardiovascular disease.

The p-values in bold denote statistical significance.

## 4 Discussion

LTL was considered as a prognostic factor in many metabolic diseases (30, 31). Aging played a key role in the course of liver fibrosis, and alcohol abuse was a major factor for liver disease progression (32). The condition of the livers of ALD patients was associated with the shortening of telomere length (33). The shrinkage of telomere contributed to cellular senescence and was recognized as a biomarker of hepatic cirrhosis separate from the kind of liver diseases including ALD (34). Furthermore, some lines of evidence supported the correlation of shortened telomeres with many liver diseases. Telomere length decreased gradually as the liver tissue deteriorated (35). Moreover, the deficiency of telomerase in liver fibrosis mouse models demonstrated a higher level of cirrhosis (36). Our research showed a negative relationship between LTL and all-cause death among ALD patients, which was consistent with a previous study (22). However, we further focused on the comprehensive impact of LTL on prognosis, including all-cause mortality, CVD, and cancer-related mortalities, and we took more covariates into consideration in prognostic models, including hematological indicators in particular. Additionally, we described the non-linear relationship between LTL and all-cause mortality as well as cancer-caused death. To the best of our knowledge, this is the first research to demonstrate L-shaped correlations between LTL and all-cause mortality. Our results indicated that LTL played an important role during the progression of liver disease, and that LTL shortening was a potential marker of abnormal liver function and worse prognosis. The corresponding comprehensive strategies should be adopted to enhance liver function according to the levels of LTL in patients with ALD so as to improve their quality of life and survival. Further studies should be performed to have a better insight into the role played by LTL in predicting mortality among ALD patients.

As was known, the abnormality of telomerase had an influence on the telomere length in various metabolic disorders, including DM, CVD, and NAFLD (14, 37, 38). Diminished LTL caused a higher risk of CVD and early death (39, 40). Shortening LTL might aggravate oxidative stress or destroy the inflammation system (41, 42). Subsequently, excess immune reaction and redundant oxidative stress were regarded as latent influencing factors for CVD (43, 44). Approximately 30% of ALD patients died from CVD in our research and longer LTL prevented patients from dying from CVD. LTL might work as a crucial predictor of cardiovascular prognosis for individuals with ALD. Moreover, relevant interventions are needed to adjust telomere length to improve patient survival. On the other hand, approximately one-third of hepatocellular cancer cases worldwide were associated with alcohol according to statistics (45). LTL shortening activated p53, which was pivotal to cell apoptosis and proliferation, and the mutation of p53 was common during carcinogenesis (46). We found that cancer-caused mortality had a higher tendency to decrease LTL. Optimized treatment strategies are needed to prove the advantage of LTL as a latent therapeutic method for ALD. More investigations are required to validate cancer-related deaths among people with ALD. In general, clinicians should examine heart function and screen tumors promptly to prevent CVD- or cancer-related death in ALD patients with shorter LTL.

Generally, women live longer than men, and gender plays various roles during the aging process (47, 48). In this study, we found that longer TLT decreased all-cause mortality especially for patients older than 65 years or male patients, which implied that sex hormones might affect the correlation between LTL and ALD. Aging was recognized as a risk factor for ALD, similar to other chronic liver diseases (49). However, there were few studies about gender differences in the aging progression. It was reported that estrogen activated telomerase (50). Men exhibited shorter life expectancies and more rapid telomere shortening. Additionally, sex-specific LTL reduction may have been a contributing factor (51, 52). Larger clinical studies are needed to confirm the role of LTL in improving survival in different genders. In addition, more laboratory experiments are needed to explore the specific mechanisms of sex hormones, LTL, and patient survival.

The increased expression of telomere binding protein possibly induced telomere shortening in patients with alcohol-associated cirrhosis (33). As a result, either the mutation of telomere-associated genes or other harmful factors might destroy the normal function of maintaining an active regenerative ability to avoid injury and fibrosis progression in the liver tissue (53, 54). Research on the influence of telomeres in ALD remained unclear, although their potential role during the ALD process was interesting. There were several opinions regarding the mechanisms about the association between LTL and liver diseases. First of all, telomerase reverse transcriptase (TERT) was an important gene for telomerase (55). Telomere shortening accelerated the devastation of liver tissue and was related to chronic inflammation. Vice versa, chronic inflammation destroyed the function of telomeres, and damaged telomeres further promoted tissue inflammation through secreting cytokine contained interleukin or TNF-α (56). The mutation of telomere-related gene (TRG) mutations significantly increased the risk of developing liver disease (57). The mutations of telomerase RNA component (TERC) sabotaged their enzymatic activity and reduced telomere length among patients with cirrhosis caused by NAFLF, ALD, or HCV infection (54). Secondly, the shortening of telomere enhanced oxidative stress by facilitating the generation of ROS and caused illnesses (58). Thirdly, shorter telomeres resulted in the dysfunction of stem cells and undermined the capacity to self-replicate, which caused tissue destruction and lowered the survival rate (59–61). More studies are needed to find the probable mechanism or molecular pathways on how alcohol influences telomere length and how telomere shortening impacts the progression of ALD.

Our study demonstrated the relationship between LTL and mortality from different aspects in patients with ALD on the basis of a large sample from NHANES 1999–2002. The robustness of our findings was confirmed by sensitivity analyses. Inevitably, there were still some limitations. First of all, we did not obtain the causation and sequence of influence between LTT and survival because of the cross-sectional and retrospective design of the study. Secondly, there might be other unknown indicators that affected the results of this study. Finally, we did not analyze how LTL affects survival in ALD due to the scarcity of more factors of liver function and the history of drug use, surgery, or liver transplantation. Further clinical investigation is warranted to substantiate this perspective. Overall, better prognosis related to LTL was a complicated field of research including various molecular mechanisms. More data and further exploration are required to evaluate a more detailed relationship between them.

## 5 Conclusion

In conclusion, our research found that LTL was an effective predictive factor for survival among patients with ALD, especially for patients >65 years or male patients. Furthermore, longer leukocyte telomere and all-cause mortality as well as cancer-related mortality had an L-shaped non-linear relationship. The causal link between LTL and survival needs to be validated by future studies to help provide effective strategies for high-risk ALD patients to prolong their life.

## Data Availability

Publicly available datasets were analyzed in this study. This data can be found here: https://www.cdc.gov/nchs/nhanes/index.htm.
