# Supplementary material for: Leukocyte telomere length decreased the risk of mortality in patients with alcohol-associated liver disease
Source: Front Endocrinol (Lausanne). 2024 Dec 12;15:1462591. doi: 10.3389/fendo.2024.1462591 (PMC11672197; doi:10.3389/fendo.2024.1462591)
Supplement: Supplementary file 1 [file Table1.docx]

Supplementary Materials

**Supplementary table 1 The associated between LTL with comorbidity profile ^a^**

| **Variables** | **Total** | **Q1** | **Q2** | **Q3** | **Q4** | **P value** |
| --- | --- | --- | --- | --- | --- | --- |
| **Hypertension** | |  |  |  |  | **<0.001** |
| No | 1591(68.29%) | 346(58.88%) | 375(65.46%) | 414(69.21%) | 456(76.65%) |  |
| Yes | 928(31.71%) | 283(41.12%) | 255(34.54%) | 217(30.79%) | 173(23.35%) |  |
| **DM** |  |  |  |  |  | **<0.001** |
| No | 2142(89.19%) | 505(84.64%) | 516(86.29%) | 554(91.72%) | 567(92.64%) |  |
| IFG | 120(3.67%) | 40(5.84%) | 32(4.62%) | 30(3.08%) | 18(1.83%) |  |
| DM | 257(7.14%) | 84(9.51%) | 82(9.09%) | 47(5.20%) | 44(5.52%) |  |
| **Anemia** |  |  |  |  |  | 0.99 |
| No | 2423(97.25%) | 597(96.94%) | 612(97.24%) | 607(97.55%) | 607(97.22%) |  |
| Yes | 95(2.75%) | 31(3.05%) | 18(2.76%) | 24(2.45%) | 22(2.78%) |  |
| Unknown | 1(0.00%) | 1(0.01%) | 0(0.00%) | 0(0.00%) | 0(0.00%) |  |
| **Cancer** |  |  |  |  |  | 0.18 |
| No | 2332(93.67%) | 554(89.65%) | 585(94.15%) | 590(93.36%) | 603(96.43%) |  |
| Yes | 186(6.33%) | 75(10.35%) | 44(5.83%) | 41(6.64%) | 26(3.57%) |  |
| Unknown | 1(0.00%) | 0(0.00%) | 1(0.02%) | 0(0.00%) | 0(0.00%) |  |
| **CKD** |  |  |  |  |  | **<0.001** |
| No | 2177(89.39%) | 491(83.75%) | 545(89.10%) | 560(90.15%) | 581(93.02%) |  |
| Yes | 333(10.23%) | 137(16.19%) | 85(10.90%) | 69(9.66%) | 42(5.88%) |  |
| Unknown | 9(0.38%) | 1(0.06%) | 0(0.00%) | 2(0.19%) | 6(1.10%) |  |
| **COPD** |  |  |  |  |  | 0.12 |
| No | 2474(98.36%) | 613(97.61%) | 618(98.10%) | 618(97.94%) | 625(99.51%) |  |
| Yes | 45(1.64%) | 16(2.39%) | 12(1.90%) | 13(2.06%) | 4(0.49%) |  |
| **Asthma** |  |  |  |  |  | 0.94 |
| No | 2198(85.35%) | 545(84.70%) | 548(85.27%) | 554(85.77%) | 551(85.51%) |  |
| Yes | 321(14.65%) | 84(15.30%) | 82(14.73%) | 77(14.23%) | 78(14.49%) |  |
| **Hyperlipidemia** | |  |  |  |  | 0.07 |
| No | 561(21.27%) | 126(17.46%) | 125(20.55%) | 146(21.41%) | 164(24.50%) |  |
| Yes | 1958(78.73%) | 503(82.54%) | 505(79.45%) | 485(78.59%) | 465(75.50%) |  |
| **CVD** |  |  |  |  |  | **<0.001** |
| No | 2314(93.46%) | 544(88.67%) | 570(91.17%) | 594(95.31%) | 606(97.21%) |  |
| Yes | 205(6.54%) | 85(11.33%) | 60(8.83%) | 37(4.69%) | 23(2.79%) |  |
| **Congestive heart failure** | | |  |  |  | 0.06 |
| No | 2466(98.45%) | 609(97.16%) | 612(97.98%) | 620(98.83%) | 625(99.44%) |  |
| Yes | 48(1.53%) | 18(2.77%) | 16(2.01%) | 10(1.17%) | 4(0.56%) |  |
| Unknown | 5(0.02%) | 2(0.07%) | 2(0.01%) | 1(0.01%) | 0(0.00%) |  |
| **PAD** |  |  |  |  |  | **<0.001** |
| No | 1460(53.83%) | 467(71.06%) | 387(57.76%) | 333(49.31%) | 273(42.12%) |  |
| Yes | 63(1.54%) | 30(3.24%) | 20(2.43%) | 10(0.68%) | 3(0.31%) |  |
| Unknown | 996(44.63%) | 132(25.70%) | 223(39.81%) | 288(50.01%) | 353(57.57%) |  |
| **Viral hepatitis** | |  |  |  |  | 0.47 |
| No | 2433(96.67%) | 607(96.52%) | 612(97.63%) | 608(95.90%) | 606(96.64%) |  |
| Yes | 86(3.33%) | 22(3.48%) | 18(2.37%) | 23(4.10%) | 23(3.36%) |  |
| **Met S** |  |  |  |  |  | **0.01** |
| No | 1811(73.77%) | 429(69.59%) | 430(70.13%) | 456(74.00%) | 496(79.67%) |  |
| Yes | 708(26.23%) | 200(30.41%) | 200(29.87%) | 175(26.00%) | 133(20.33%) |  |
| **BMI** | 28.44(0.13%) | 29.01(0.24%) | 28.84(0.30%) | 28.36(0.21%) | 27.75(0.26%) | **0.01** |

^a^ Data were adjusted for NHANES survey weights. IFG, impaired fasting glucose; DM, diabetes mellitus; CKD, chronic kidney disease; COPD, chronic obstructive pulmonary disease; CVD, cerebrovascular diseases; PAD, peripheral arterial disease; Met S, metabolic syndrome.

**Supplementary table 2** The associated between LTL with mortality regarded as a continuous variable **^a^**

| **character** | Model 1 | | | Model 2 | | | Model 3 | | | Model 4 | | |
| --- | --- | --- | --- | --- | --- | --- | --- | --- | --- | --- | --- | --- |
|  | **HR** | **95% CI** | **P value** | **HR** | **95% CI** | **P value** | **HR** | **95% CI** | **P value** | **HR** | **95% CI** | **P value** |
| **All causes** | 0.11 | 0.06-0.20 | **＜0.001** | 0.31 | 0.18-0.52 | **＜0.001** | 0.34 | 0.20-0.56 | **＜0.001** | 0.37 | 0.23-0.61 | **＜0.001** |
| **CVD** | 0.03 | 0.01-0.10 | **＜0.001** | 0.08 | 0.03-0.22 | **＜0.001** | 0.09 | 0.03-0.24 | **＜0.001** | 0.09 | 0.03-0.26 | **＜0.001** |
| **Cancer** | 0.25 | 0.06-0.95 | **0.04** | 0.46 | 0.14-1.52 | 0.2 | 0.51 | 0.14-1.90 | 0.32 | 0.58 | 0.16-2.08 | 0.41 |

^a^ Data were adjusted for NHANES survey weights. Model 1 was not adjusted for any covariates. Model 2 was adjusted for age, gender and race. Model 3 was further adjusted for education, smoke, hypertension and DM on the basis of model 2. Model 4 was adjusted for other laboratory indicators, including PIR, Albumin(g/L), CRP (mg/dl), ALT (U/L), AST (U/L), ALP (U/L), Total Bilirubin (mg/dl) and LDH (U/L). Q1, quartile 1; Q2, quartile 2; Q3, quartile 3; Q4, quartile 4; HR, hazard ratio; CI, confidence interval; CVD, cardiovascular disease.

**Supplementary table 3 The association between LTL and mortality after excluding individuals died within two-year-follow-up ^a^**

| **Character** | **HR (95% CI)** | **P value** | **HR (95% CI)** | **P value** | **HR (95% CI)** | **P value** | **HR (95% CI)** | **P value** |
| --- | --- | --- | --- | --- | --- | --- | --- | --- |
| **All causes** |  |  |  |  |  |  |  |  |
| Q1 | - | - | - | - | - | - | - | - |
| Q2 | 0.54(0.41-0.71) | **<0.0001** | 0.72(0.54-0.95) | **0.02** | 0.69(0.53-0.90) | **0.01** | 0.72(0.55-0.93) | **0.01** |
| Q3 | 0.40(0.29-0.55) | **<0.0001** | 0.63(0.43-0.91) | **0.01** | 0.62(0.44-0.88) | **0.01** | 0.67(0.47-0.96) | **0.03** |
| Q4 | 0.25(0.19-0.34) | **<0.0001** | 0.43(0.32-0.59) | **<0.0001** | 0.46(0.33-0.64) | **<0.0001** | 0.49(0.36-0.67) | **<0.0001** |
| **CVD** |  |  |  |  |  |  |  |  |
| Q1 | - | - | - | - | - | - | - | - |
| Q2 | 0.51(0.35-0.76) | **<0.0001** | 0.67(0.42-1.07) | 0.09 | 0.67(0.41-1.08) | 0.1 | 0.68(0.42-1.09) | 0.11 |
| Q3 | 0.26(0.14-0.49) | **<0.0001** | 0.40(0.22-0.70) | **0.002** | 0.42(0.24-0.73) | **0.002** | 0.45(0.26-0.79) | **0.01** |
| Q4 | 0.11(0.05-0.22) | **<0.0001** | 0.17(0.09-0.35) | **<0.0001** | 0.19(0.09-0.38) | **<0.0001** | 0.20(0.10-0.39) | **<0.0001** |
| **Cancer** |  |  |  |  |  |  |  |  |
| Q1 | - | - | - | - | - | - | - | - |
| Q2 | 0.37(0.18-0.76) | **0.01** | 0.44(0.22-0.88) | **0.02** | 0.48(0.25-0.90) | **0.02** | 0.48(0.25-0.92) | **0.03** |
| Q3 | 0.55(0.29-1.03) | 0.06 | 0.76(0.40-1.43) | 0.39 | 0.83(0.44-1.57) | 0.57 | 0.89(0.47-1.69) | 0.72 |
| Q4 | 0.31(0.16-0.61) | **<0.0001** | 0.45(0.24-0.85) | **0.01** | 0.49(0.25-0.96) | **0.04** | 0.52(0.27-1.02) | 0.06 |

^a^ Data were adjusted for NHANES survey weights. Model 1 was not adjusted for any covariates. Model 2 was adjusted for age, gender and race. Model 3 was further adjusted for education, smoke, hypertension and DM on the basis of model 2. Model 4 was adjusted for other laboratory indicators, including PIR, Albumin(g/L), CRP (mg/dl), ALT (U/L), AST (U/L), ALP (U/L), Total Bilirubin (mg/dl) and LDH (U/L). Q1, quartile 1; Q2, quartile 2; Q3, quartile 3; Q4, quartile 4; HR, hazard ratio; CI, confidence interval; CVD, cardiovascular disease.

**Supplementary table 4 The association between LTL and mortality after excluding individuals with cancer ^a^**

| **Character** | **HR (95% CI)** | **P value** | **HR (95% CI)** | **P value** | **HR (95% CI)** | **P value** | **HR (95% CI)** | **P value** |
| --- | --- | --- | --- | --- | --- | --- | --- | --- |
| **All causes** |  |  |  |  |  |  |  |  |
| Q1 | - | - | - | - | - | - | - | - |
| Q2 | 0.57(0.44-0.74) | **<0.0001** | 0.73(0.58-0.93) | **0.01** | 0.71(0.56-0.90) | **0.01** | 0.72(0.57-0.91) | **0.01** |
| Q3 | 0.47(0.33-0.68) | **<0.0001** | 0.69(0.47-1.02) | 0.07 | 0.72(0.49-1.05) | 0.09 | 0.76(0.52-1.11) | 0.16 |
| Q4 | 0.27(0.19-0.37) | **<0.0001** | 0.42(0.29-0.59) | **<0.0001** | 0.46(0.32-0.66) | **<0.0001** | 0.48(0.34-0.68) | **<0.0001** |
| **CVD** |  |  |  |  |  |  |  |  |
| Q1 | - | - | - | - | - | - | - | - |
| Q2 | 0.48(0.31-0.75) | **0.001** | 0.64(0.40-1.03) | 0.06 | 0.66(0.40-1.10) | 0.11 | 0.66(0.40-1.09) | 0.1 |
| Q3 | 0.28(0.16-0.52) | **<0.0001** | 0.43(0.25-0.73) | **0.002** | 0.46(0.27-0.78) | **0.004** | 0.50(0.29-0.85) | **0.01** |
| Q4 | 0.12(0.06-0.27) | **<0.0001** | 0.19(0.09-0.40) | **<0.0001** | 0.21(0.09-0.47) | **<0.0001** | 0.22(0.10-0.47) | **<0.0001** |
| **Cancer** |  |  |  |  |  |  |  |  |
| Q1 | - | - | - | - | - | - | - | - |
| Q2 | 0.47(0.23-0.93) | **0.03** | 0.54(0.28-1.05) | 0.07 | 0.57(0.32-1.04) | 0.07 | 0.58(0.31-1.12) | 0.11 |
| Q3 | 0.69(0.36-1.32) | 0.26 | 0.89(0.47-1.68) | 0.71 | 0.99(0.51-1.89) | 0.97 | 1.05(0.54-2.03) | 0.88 |
| Q4 | 0.32(0.17-0.60) | **<0.0001** | 0.42(0.23-0.76) | **0.005** | 0.48(0.25-0.92) | **0.03** | 0.50(0.27-0.96) | **0.04** |

^a^ Data were adjusted for NHANES survey weights. Model 1 was not adjusted for any covariates. Model 2 was adjusted for age, gender and race. Model 3 was further adjusted for education, smoke, hypertension and DM on the basis of model 2. Model 4 was adjusted for other laboratory indicators, including PIR, Albumin(g/L), CRP (mg/dl), ALT (U/L), AST (U/L), ALP (U/L), Total Bilirubin (mg/dl) and LDH (U/L). Q1, quartile 1; Q2, quartile 2; Q3, quartile 3; Q4, quartile 4; HR, hazard ratio; CI, confidence interval; CVD, cardiovascular disease.

**Supplementary table 5 The association between LTL and mortality when the analysis didn’t been adjusted for NHANES survey weights**

| **Character** | **HR (95% CI)** | **P value** | **HR (95% CI)** | **P value** | **HR (95% CI)** | **P value** | **HR (95% CI)** | **P value** |
| --- | --- | --- | --- | --- | --- | --- | --- | --- |
| **All causes** |  |  |  |  |  |  |  |  |
| Q1 | - | - | - | - | - | - | - | - |
| Q2 | 0.50(0.40-0.61) | **<0.0001** | 0.67(0.54-0.83) | **<0.0001** | 0.63(0.51-0.78) | **<0.0001** | 0.64(0.52-0.80) | **<0.0001** |
| Q3 | 0.37(0.29-0.46) | **<0.0001** | 0.62(0.49-0.79) | **<0.0001** | 0.62(0.49-0.79) | **<0.0001** | 0.66(0.52-0.84) | **<0.0001** |
| Q4 | 0.26(0.20-0.33) | **<0.0001** | 0.48(0.37-0.64) | **<0.0001** | 0.52(0.39-0.68) | **<0.0001** | 0.54(0.41-0.71) | **<0.0001** |
| **CVD** |  |  |  |  |  |  |  |  |
| Q1 | - | - | - | - | - | - | - | - |
| Q2 | 0.53(0.36-0.80) | **0.002** | 0.72(0.48-1.08) | 0.11 | 0.64(0.43-0.97) | **0.04** | 0.61(0.40-0.92) | **0.02** |
| Q3 | 0.31(0.20-0.50) | **<0.0001** | 0.53(0.33-0.87) | **0.01** | 0.55(0.34-0.89) | **0.02** | 0.53(0.33-0.87) | **0.01** |
| Q4 | 0.15(0.08-0.28) | **<0.0001** | 0.29(0.15-0.55) | **<0.0001** | 0.31(0.16-0.59) | **<0.0001** | 0.30(0.16-0.56) | **<0.0001** |
| **Cancer** |  |  |  |  |  |  |  |  |
| Q1 | - |  |  | - |  | - | - | - |
| Q2 | 0.34(0.22-0.54) | **<0.0001** | 0.43(0.27-0.68) | **<0.0001** | 0.42(0.27-0.67) | **<0.0001** | 0.44(0.27-0.70) | **<0.0001** |
| Q3 | 0.41(0.27-0.62) | **<0.0001** | 0.62(0.40-0.96) | **0.03** | 0.66(0.43-1.02) | 0.06 | 0.73(0.47-1.13) | 0.15 |
| Q4 | 0.28(0.18-0.45) | **<0.0001** | 0.47(0.28-0.76) | **0.002** | 0.49(0.30-0.80) | **0.004** | 0.52(0.32-0.85) | **0.01** |

Model 1 was not adjusted for any covariates. Model 2 was adjusted for age, gender and race. Model 3 was further adjusted for education, smoke, hypertension and DM on the basis of model 2. Model 4 was adjusted for other laboratory indicators, including PIR, Albumin(g/L), CRP (mg/dl), ALT (U/L), AST (U/L), ALP (U/L), Total Bilirubin (mg/dl) and LDH (U/L). Q1, quartile 1; Q2, quartile 2; Q3, quartile 3; Q4, quartile 4; HR, hazard ratio; CI, confidence interval; CVD, cardiovascular disease.
